# Placental Syncytiotrophoblast Constitutes a Major Barrier to Vertical Transmission of *Listeria monocytogenes*

**DOI:** 10.1371/journal.ppat.1000732

**Published:** 2010-01-22

**Authors:** Jennifer R. Robbins, Kasia M. Skrzypczynska, Varvara B. Zeldovich, Mirhan Kapidzic, Anna I. Bakardjiev

**Affiliations:** 1 Department of Pediatrics, University of California, San Francisco, California, United States of America; 2 Program in Microbial Pathogenesis and Host Defense, University of California, San Francisco, California, United States of America; 3 Department of Biology, Xavier University, Cincinnati, Ohio, United States of America; 4 Institute for Regeneration Medicine, Human Embryonic Stem Cell Program, Department of Obstetrics and Gynecology, University of California, San Francisco, California, United States of America; Stanford University, United States of America

## Abstract

*Listeria monocytogenes* is an important cause of maternal-fetal infections and serves as a model organism to study these important but poorly understood events. *L. monocytogenes* can infect non-phagocytic cells by two means: direct invasion and cell-to-cell spread. The relative contribution of each method to placental infection is controversial, as is the anatomical site of invasion. Here, we report for the first time the use of first trimester placental organ cultures to quantitatively analyze *L. monocytogenes* infection of the human placenta. Contrary to previous reports, we found that the syncytiotrophoblast, which constitutes most of the placental surface and is bathed in maternal blood, was highly resistant to *L. monocytogenes* infection by either internalin-mediated invasion or cell-to-cell spread. Instead, extravillous cytotrophoblasts—which anchor the placenta in the decidua (uterine lining) and abundantly express E-cadherin—served as the primary portal of entry for *L. monocytogenes* from both extracellular and intracellular compartments. Subsequent bacterial dissemination to the villous stroma, where fetal capillaries are found, was hampered by further cellular and histological barriers. Our study suggests the placenta has evolved multiple mechanisms to resist pathogen infection, especially from maternal blood. These findings provide a novel explanation why almost all placental pathogens have intracellular life cycles: they may need maternal cells to reach the decidua and infect the placenta.

## Author Summary

Placental infections can lead to severe pregnancy complications as well as infection of the fetus and newborn with significant morbidity and mortality. Pathogens that are able to cross the maternal-fetal barrier typically have life cycles inside host cells. Among these is the facultative intracellular bacterial pathogen *Listeria monocytogenes*, which is highly amenable to experimental analysis. Our study is the first to use early gestation primary human placental organ cultures to identify the mechanisms by which *L. monocytogenes* breaches the human maternal-fetal barrier. We found that the placenta has evolved multiple mechanisms to resist infection. The main portal of entry into the placenta was a small subpopulation of fetally derived trophoblast cells (extravillous cytotrophoblasts), which anchor the placenta in the decidua, the lining of the pregnant uterus. These cells could be infected via two mechanisms: direct invasion of extracellular bacteria and cell-to-cell spread. The extravillous cytotrophoblasts are not readily accessible from the maternal blood stream. This is a significant finding because it provides a novel explanation why almost all placental pathogens have intracellular life cycles: they may need maternal cells to reach the decidua and infect the placenta.

## Introduction

Infection is a major cause for pregnancy complications including premature labor and resultant maternal and fetal morbidity and mortality (WHO, 2005). Nevertheless, the underlying mechanisms of placental and fetal infection are poorly understood. The placenta and fetus are vulnerable to infection via two different routes: (a) pathogens in the lower genital tract may ascend through the cervix and (b) pathogens in the maternal blood or uterus can colonize the placenta and breach the maternal-fetal barrier. The later group includes many viruses, e.g. cytomegalovirus; protozoan parasites, e.g. *Toxoplasma gondii*; and bacterial pathogens, e.g. *Listeria monocytogenes*. It is striking that the majority of pathogens that are able to cross the placenta have either facultative or obligate intracellular life cycles. The reason for the predisposition of placental infection toward intracellular pathogens is unclear. It has been postulated that the unique immunological environment in the placenta—necessary to assure tolerance of the fetal allograft—contributes to this phenomenon [1]–[3], but other aspects of the placenta may also play a role.

*L. monocytogenes* is a ubiquitous bacterial pathogen that causes food-borne disease in humans and many other mammals [4]–[6]. In pregnant women *L. monocytogenes* can spread to the placenta and fetus, resulting in spontaneous abortion, stillbirth, or preterm labor, depending on the gestational age [7]. The incidence of *L. monocytogenes*-induced spontaneous abortion during the first trimester is unknown; such early abortions are often due to chromosomal abnormalities [8] and therefore the aborted tissues are not routinely cultured. During the second trimester, *L. monocytogenes* has been found to cause ∼3% of spontaneous abortions in humans and cattle [9]–[11]. Clinical infections of the mother at term are rare, but when they occur, they can result in neonatal disease with mortality of up to 50% [12].

Among the intracellular microbes known to cross the maternal-fetal barrier, *L. monocytogenes* is particularly amenable to experimental analysis. *L. monocytogenes* has been used for decades as a model system to evaluate intracellular pathogenesis and the host's cell mediated and innate immune response to infection (for recent reviews see [13]–[15]). *L. monocytogenes* can infect professional phagocytic and non-phagocytic cells in many species. A family of bacterial cell wall surface proteins called internalins (Inl) promote bacterial adherence and internalization into non-phagocytic host cells [16]. Of these, internalin A (InlA) and internalin B (InlB) are the best characterized, binding to E-cadherin and c-Met-tyrosine kinase, respectively [17],[18]. After internalization, the bacterium escapes from the vacuole into the host cell cytoplasm where it multiplies rapidly [19],[20]. The listerial virulence determinant ActA facilitates spread from infected host cells to neighboring cells without bacterial exposure to the extracellular environment [21]–[24]. Thus, *L. monocytogenes* is able to infect non-phagocytic cells by two different mechanisms: Inl-mediated direct invasion and cell-to-cell spread. In the work described herein, we determine the placental tissue barriers operative against each mechanism and explore how *L. monocytogenes* might overcome them.

**Figure 1:**
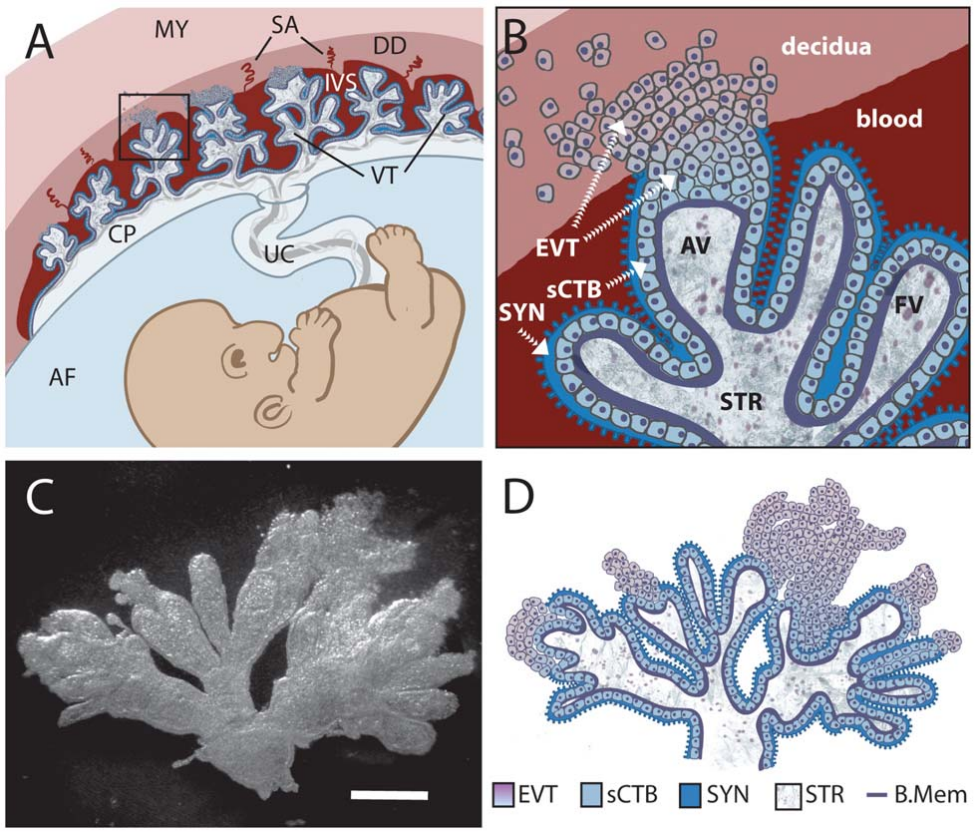
Comparison of in vivo placental structure to placenta explant model. (**A**) Structure and orientation of fetus and placenta in uterus at ∼6 weeks of gestation. Fetal structures are represented in shades of blue and purple while maternal are in shades of red. Maternal structures: MY: myometrium, SA: spiral arteries, DD: decidua (uterine lining during pregnancy), IVS: intervillous space filled with maternal blood. Fetal structures: VT: villous tree, CP: chorionic plate, UC: umbilical cord, AF: amniotic fluid. (**B**) (Enlargement of boxed area in panel A) Maternal blood surrounds the villous tree composed of anchoring (AV) and floating villi (FV), which are covered by a syncytiotrophoblast (SYN) that is underlaid by subsyncytial cytotrophoblasts (sCTB) and a basement membrane. The subsyncytial CTB layer grows increasingly discontinuous in later trimesters. Gas and nutrient exchange with the maternal blood occurs across the syncytiotrophoblast to supply fetal capillaries in the stroma (STR). At the uterine wall, extravillous cytotrophoblasts (EVT) anchor the villous tree in the decidua. Some invade the decidua and move away from the tip to remodel maternal spiral arteries, with altered gene expression patterns as they move (not shown). Notably, E-cadherin expression decreases as VE-cadherin expression rises in distal (relative to fetus) extravillous cytotrophoblasts. (**C**) A six-week placental explant anchored in Matrigel. Bar = 1 mm. (**D**) Cartoon representation of the relevant structures seen in panel C.

In order to understand the mechanisms leading to placental and fetal infection it is essential to understand the structure and physiology of the placenta. The placenta is made of maternal and fetal tissues. Placentas of different viviparous vertebrates exhibit great variability at the maternal-fetal interface, complicating cross-species comparisons [25]. Humans have a hemomonochorial villous placenta (Figure 1A). Maternal blood from spiral arteries in the decidua (uterine lining during pregnancy) flows into the intervillous space where it surrounds thousands of fetally derived floating villi. Some villi invade the decidua and form anchoring villi. The entire villous surface is covered with a continuous layer of multinucleate syncytiotrophoblast (SYN) (Figure 1B), which is the major fetal surface in contact with maternal blood. The apical side of the syncytium consists of profuse, branched microvilli [26],[27] and provides abundant surface area for gas and nutrient exchange between mother and fetus. The syncytiotrophoblast is undergirded by cytotrophoblasts (CTB) [28], which are separated from fetal capillaries in the villous stroma by a basement membrane. Some cytotrophoblasts leave the basement membrane and differentiate along the invasive pathway to form anchoring villi: columns of unpolarized cytotrophoblasts attach to and then penetrate the uterine wall where they give rise to extravillous cytotrophoblasts [29]. Extravillous cytotrophoblasts commingle with resident decidual, myometrial and immune cells. A subset of extravillous cytotrophoblasts breaches maternal spiral arteries in the decidua and differentiates into endovascular trophoblasts that replace the resident maternal endothelium to direct more blood into the intervillous space [29].

The anatomical site and mechanism by which *L. monocytogenes* breaches the maternal-fetal barrier are controversial. Of particular interest is whether InlA-mediated binding to E-cadherin is essential for transplacental transmission. Infection of isolated human cytotrophoblasts [30] or the BeWo choriocarcinoma cell line [31] with *L. monocytogenes* deficient in InlA leads to a 100-fold reduction in invasion. However, *in vivo*, cytotrophoblasts are covered with syncytiotrophoblast and may not be accessible to the bacteria. Lecuit et al. reported that E-cadherin is expressed at low levels on the apical surface of syncytiotrophoblast in explants from human term placentas [31], and postulated that *L. monocytogenes* breaches the maternal-fetal barrier by InlA-mediated invasion of the syncytiotrophoblast from the maternal bloodstream [31]. However, other groups have not observed E-cadherin expression on the surface of the syncytium [32]–[36]. Furthermore, InlA or InlB mutants do not affect feto-placental infection in guinea pigs [30] (unpublished observations), and show less than a 5-fold decrease in bacterial numbers in the gerbil placenta and fetus [37]. Wild type InlA does not interact with murine E-cadherin [38], but infection of wild type mice with *L. monocytogenes* expressing murinized InlA does not influence the course of feto-placental listeriosis [39], and in transgenic mice expressing human E-cadherin, InlA and/or InlB have a <5-fold effect on placental and fetal infection [37]. The minimal or absent *in vivo* phenotype observed with internalin mutants in these four rodent models is surprising given the strong phenotype in isolated cytotrophoblasts and suggests that the syncytiotrophoblast may not be the initial site of infection.

InlA/E-cadherin is not the only mechanism for infection—*L. monocytogenes* can spread from cell-to-cell without exposure to the extracellular environment, and there is evidence that *L. monocytogenes* traffics to the placenta [40] or the brain [41] inside of cells. Furthermore, we and others have found cell-to-cell spread to be important for fetal infection [42],[43].

In this report, we probe the human maternal-fetal barrier using first trimester human placental organ cultures, which allow a detailed examination of the most likely sites of transplacental infection by direct incubation with extracellular *L. monocytogenes* as well as via co-incubation with infected human cells. We found intact syncytiotrophoblast to be resistant to infection by *L. monocytogenes*. The portal of entry for *L. monocytogenes* was instead a small subpopulation of E-cadherin-expressing extravillous cytotrophoblasts in anchoring villi that are not readily accessible from the maternal bloodstream *in vivo*, and infection of these cells occurred via both InlA-mediated invasion and cell-to-cell spread. Surprisingly, these cells were able to restrict the growth of *L. monocytogenes*. If infection progressed, the bacteria spread along subsyncytial cytotrophoblasts, mostly sparing the syncytiotrophoblast and villous stroma. Our results clarify the mechanisms of crossing the maternal-fetal barrier and provide a unifying explanation for the conflicting *in vitro* and *in vivo* results mentioned above.

## Results

### Culture of first trimester human placental explants

In order to examine the role of direct invasion and cell-to-cell spread in breaching the human maternal-fetal barrier we turned to first trimester human placental organ cultures, a well-studied model system that allows examination of the trophoblast in a context that retains the cellular architecture of the tissue *in vivo* [44],[45]. Placental villous trees are dissected and explanted on substrates of extracellular matrix (Matrigel), where they form floating and anchoring villi (Figure 1C-D). All of the tissue is exposed to the media with the exception of the tips of anchoring villi that result from extravillous cytotrophoblast outgrowth and invasion into Matrigel [45],[46]. This mimics the conditions *in vivo* where extravillous cytotrophoblasts invade the decidua while the rest of the villous tree is bathed in maternal blood [29]. The syncytiotrophoblast covers the villi and remains largely intact for at least 24 h (Susan Fisher, personal communication). First trimester placental explants therefore adequately represent the most probable placental sites that are potentially accessible to *L. monocytogenes* or infected phagocytes: intact syncytiotrophoblast, subsyncytial cytotrophoblasts underlying damaged syncytiotrophoblast, and extravillous cytotrophoblasts (Figure 1).

### *L. monocytogenes* infection of human placental explants

**Figure 2:**
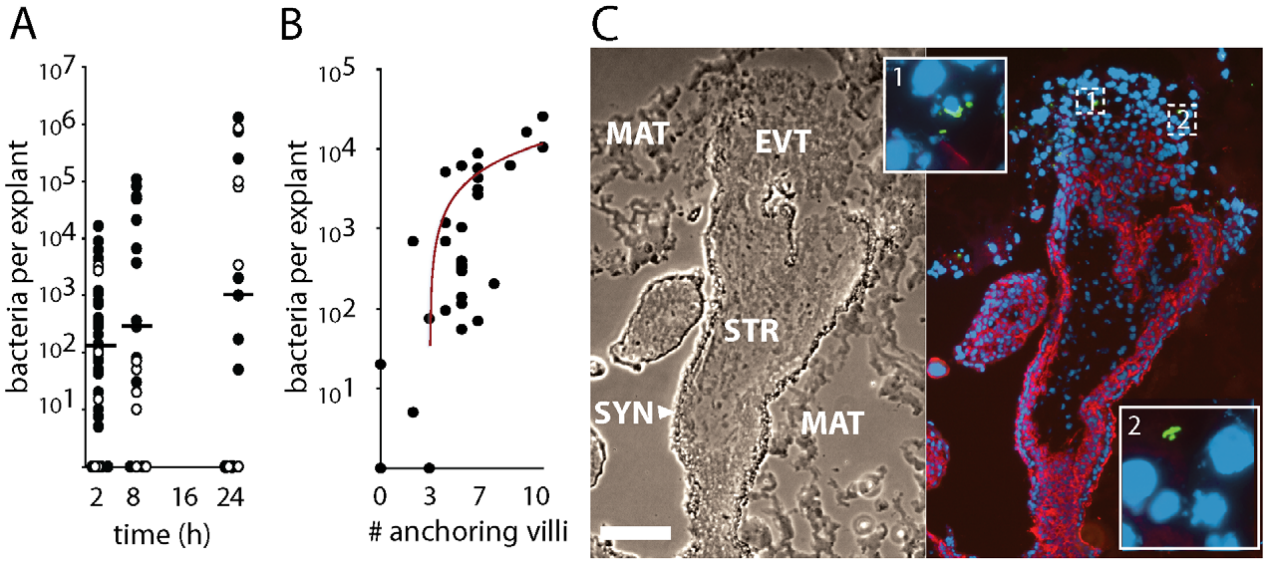
*L. monocytogenes* grows variably in placental explants. (**A**) Intracellular survival of *L. monocytogenes* in 86 explants from 18 placentas infected with ∼2×10^6^ 10403S (filled circles) or EGDe (open circles) wild type strains for 30 min. Gentamicin was added at 60**min to kill extracellular bacteria and maintained in media thereafter. Infection is highly variable and growth is slower than in most cell lines. Bars = median values. (**B**) Number of internalized bacteria at 2 h post inoculation (p.i.) correlates with the number of anchoring villi in the explant (n = 30 explants, r^2^ = 0.49). (**C**) Histological section of explant frozen and sliced at 8 h p.i., then stained for *L. monocytogenes* (green), DNA (blue), and EGFR (red), which stains trophoblast membranes. Bacteria are found in extravillous cytotrophoblasts (EVT) but not syncytiotrophoblast (SYN). Matrigel (MAT) and stroma (STR) are also indicated. Bar = 100 µm.

We infected explants with 2×10^6^ wild type *L. monocytogenes* (Figure 2). In order to measure intracellular growth we added gentamicin 1 h post-inoculation to eliminate extracellular *L. monocytogenes*, and subsequently determined numbers of live bacteria per explant over 24 h (Figure 2A). No significant differences in infection were observed between the wild type strains 10403S and EGDe (p = 0.38 by Student's T-test). The average bacterial growth from all placentas was less than 10-fold, which is slow compared to that found in cell lines [20] (Figure 2A), and infection rates were highly variable. Despite the relatively high inoculum, 11% of the explants were not infected and an additional 13% hosted <10 intracellular bacteria at 2 h post-inoculation. The average percentage of intracellular bacteria at 2 h post-inoculation was 0.6%±2% SD of the inoculum (n = 54 explants), similar to the bottleneck in the pregnant guinea pig model of listeriosis [40].

Explants vary in size, shape, age, donor and degree of Matrigel invasion, so variability is expected. However, we were able to distinguish two possible courses of infection by examining three explants from the same placenta at each time-point. Roughly half of the placentas exhibited an increase in bacterial numbers from 2 to 24 h (average = 77-fold, SD = 6.4) while the others showed a decrease (average = 0.25-fold, SD = 0.19).

It has been previously suggested that *L. monocytogenes* invades the syncytiotrophoblast [31]. If this is true for explants, then larger explants should be more highly infected, since >90% of each explant's bacterially accessible surface area is covered by syncytiotrophoblast. But we found no correlation between colony forming units (CFU) and explant size (r^2^<0.05) in 30 explants from 11 placentas. Nor did explant age affect CFU (r^2^<0.02). However, CFU at 2 h post-inoculation did correlate with the number of anchoring villi (r^2^ = 0.49, Figure 2B), suggesting that extravillous cytotrophoblasts are the preferred sites of *L. monocytogenes* infection. Examination by immunofluorescence histology revealed only a few foci of infection, usually in extravillous cytotrophoblasts of anchoring villi (Figure 2C).

### Syncytiotrophoblast forms a barrier against infection

**Figure 3:**
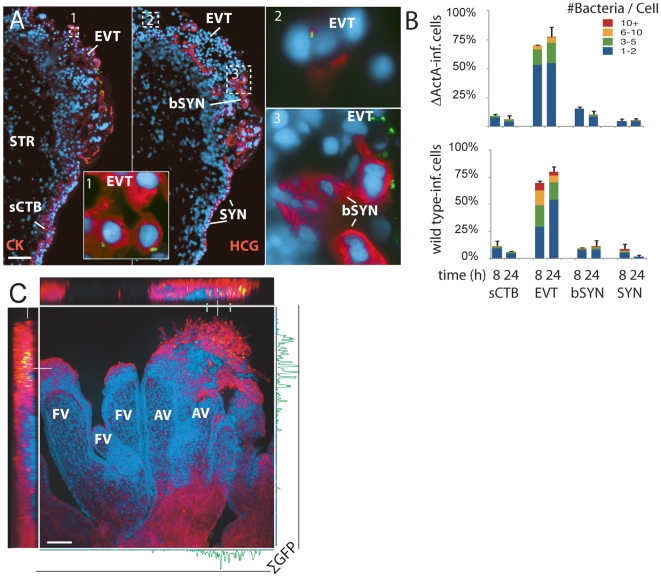
*L. monocytogenes* enters the placenta primarily at invasive villus tips. (**A**) Consecutive histological sections of a permissively infected explant at 8 h post-inoculation, frozen and stained for *L. monocytogenes* (green) and DNA (blue). Left panel and inset 1: red = cytokeratin (CK), expressed by cytotrophoblasts (CTB). In middle panel with insets 2 and 3: red = βHCG (HCG), which primarily stains syncytiotrophoblast (SYN). Subsyncytial cytotrophoblasts (sCTB) underlie the syncytiotrophoblast. Where cytotrophoblasts invade from the villus into the decidua, syncytiotrophoblast breaks, exposing basal surfaces (bSYN). Scattered, isolated bacteria are found mainly in proximal extravillous cytotrophoblasts (EVT). Bar = 100 µm. (**B**) Distribution of infected cell types in explants infected with ΔActA (top) or 10403S wild type *L. monocytogenes* (bottom). Each graph represents two sections in each of three explants (average of infected cells counted per explant = 135). For SYN and bSYN, a “cell” was considered to be the area around a single nucleus, roughly the size of a cytotrophoblast. Bars are SEM. (**C**) Projection of a 3D confocal image showing a whole explant permissively infected with GFP-expressing *L. monocytogenes* and fixed at 8 h. Anchoring villi (AV), which include invading extravillous cytotrophoblasts, and floating villi (FV), which remain covered with syncytiotrophoblast, are indicated. Red = F-actin. Green = *L. monocytogenes*. Blue = DNA. Left and top: reconstructed Z series. Because of high F-actin levels in extravillous cytotrophoblasts, bacteria appear yellow. Right and bottom: sum of total GFP intensity over 70 µm Z stack for each X/Y position after background subtraction shows the majority of bacteria are in anchoring villi, in extravillous cytotrophoblasts. Bar = 100 µm.

To better characterize which placental cell types are most vulnerable to *L. monocytogenes* infection, we increased both the inoculum and the time of incubation without gentamicin. These “permissive infections” increase the probability of infection at vulnerable sites. In addition to wild type bacteria, we also used bacteria deficient in ActA (ΔActA) that are incapable of intercellular spread, thus ensuring that the *L. monocytogenes*-containing cells we observe are those initially infected by the bacteria. After 8 h, we examined explant sections by immunofluorescence (Figure 3).

Under these conditions, *L. monocytogenes* was detectable in three cell types (Figure 3A): subsyncytial cytotrophoblasts, extravillous cytotrophoblasts and syncytiotrophoblast. Infected syncytiotrophoblast could be subdivided into: 1) apparently intact syncytiotrophoblast where only the apical surface is exposed to bacteria; and 2) basolaterally accessible syncytiotrophoblast (bSYN), where the syncytiotrophoblast is naturally terminated by an invading CTB cell column (Figure 3A) or, in rare cases, torn away from the explant, presumably during dissection.

We enumerated the total number of infected cells in explant sections (Figure 3B). For syncytiotrophoblast, a “cell” was defined as a circular region similar in size to a CTB, roughly the area surrounding a single nucleus. Infection of subsyncytial cytotrophoblasts was infrequent, which is unsurprising since unlike syncytiotrophoblast and extravillous cyotrophoblasts they are largely inaccessible to bacteria in the media. However, syncytiotrophoblast infection was also low, even though it covers almost all of the explant surface. Roughly 75% of the infected cells were extravillous cytotrophoblasts, which comprise less than 5% of available surface. Furthermore, these cells were ∼5 times more likely to contain multiple bacteria, possibly indicating multiple infections.

Transverse sections obscure a full view of the syncytiotrophoblast. To ensure that our observations were not a histological artifact, we fixed and mounted whole explants infected with *L. monocytogenes* expressing GFP. Confocal microscopy of these minimally manipulated explants confirmed that the bacteria are highly localized within the extravillous cytotrophoblasts of anchoring villi (Figure 3C). Together, these results suggest that extravillous cytotrophoblasts may serve as the primary site of infection.

Although in cultured macrophage and epithelial cell lines cell-to-cell spread begins around 4–5 h post-infection [21],[24], we found no significant difference between locations of ΔActA and wild type *L. monocytogenes* at 8 or even 24 h (Figure 3B, p = 0.99 by chi-squared test), suggesting that the *L. monocytogenes* life cycle (intracellular growth and/or cell-to-cell spread) is delayed in primary trophoblast cells.

### InlA mediates proximal extravillous cytotrophoblast invasion

**Figure 4:**
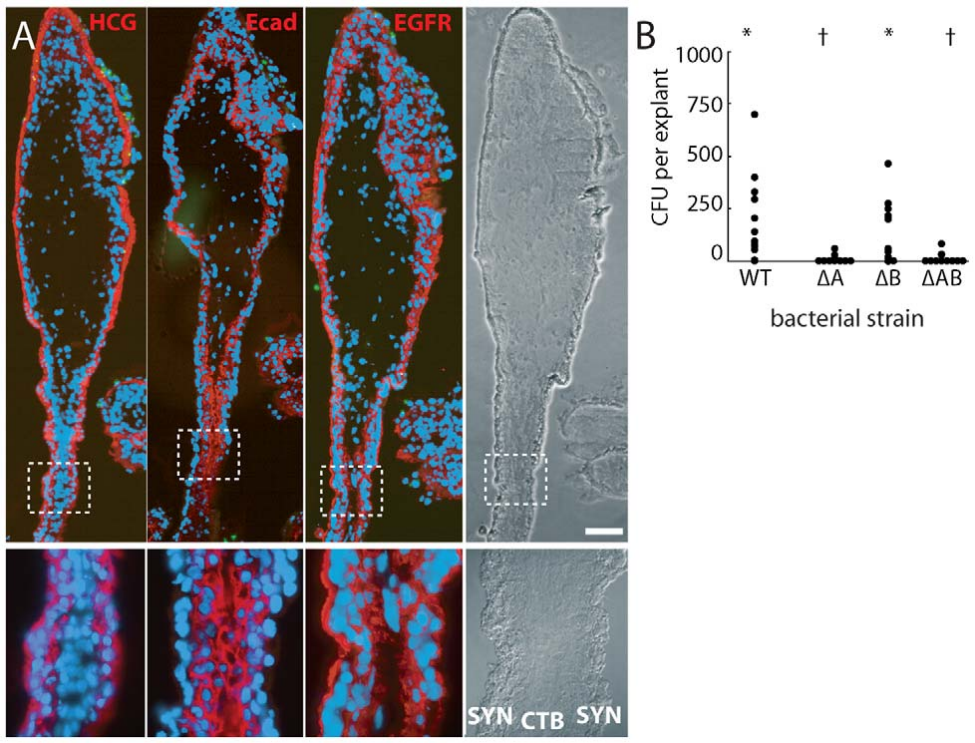
Bacteria invade primarily via InlA binding to E-cadherin on cytotrophoblasts not covered by syncytiotrophoblast. (**A**) Immunofluorescence of consecutive histological sections. From left to right: red stains βHCG (HCG, a syncytiotrophoblast marker), E-cadherin (Ecad) and EGFR (stains cytotrophoblasts (CTB) and syncytiotrophoblast (SYN) membrane). E-cadherin does not appear on the apical surface of syncytiotrophoblast but is abundant in cytotrophoblasts. Green = *L. monocytogenes.* Blue = DNA. Bar = 100 µm. (**B**) Intracellular invasion of *L. monocytogenes* strains deficient in InlA (ΔA), InlB (ΔB), or InlA/InlB (ΔAB) at 2 h post-inoculation. Each condition represents at least 4 placentas and 3 explants per placenta. Asterisks and crosses denote statistically similar populations.

E-cadherin is an important host cell receptor for *L. monocytogenes* binding and uptake. Lecuit et al. suggested that *L. monocytogenes* extracellular invasion of the placenta occurs via *L. monocytogenes* InlA interactions with host E-cadherin on the apical surface of syncytiotrophoblast [31]. However, other studies of the placenta have failed to find E-cadherin here [32]–[36]. Our results support this: we never observed E-cadherin staining on the apical surface of the syncytiotrophoblast, although it was expressed strongly on the basal surface (Figure 4A). Like others, we found E-cadherin was most abundant on subsyncytial cytotrophoblasts and proximal extravillous cytotrophoblasts, decreasing as cells migrate away from the villus tip.

Since proximal extravillous cytotrophoblasts were the very cells *L. monocytogenes* infected, we hypothesized that explant infection is InlA-dependent. Indeed, ΔInlA and ΔInlAB mutants were almost completely unable to invade explants (Figure 4B; *p*<10^−20^ by Student's T-test). We found no significant difference between invasion of wild type and ΔInlB *L. monocytogenes* in human explants (Figure 4B; *p* = 0.68 by Student's T-test) consistent with previous observations in isolated CTB and BeWo cells (human choriocarcinoma cell line) [30].

### Damage of syncytiotrophoblast leads to infection of subsyncytial cytotrophoblasts

**Figure 5:**
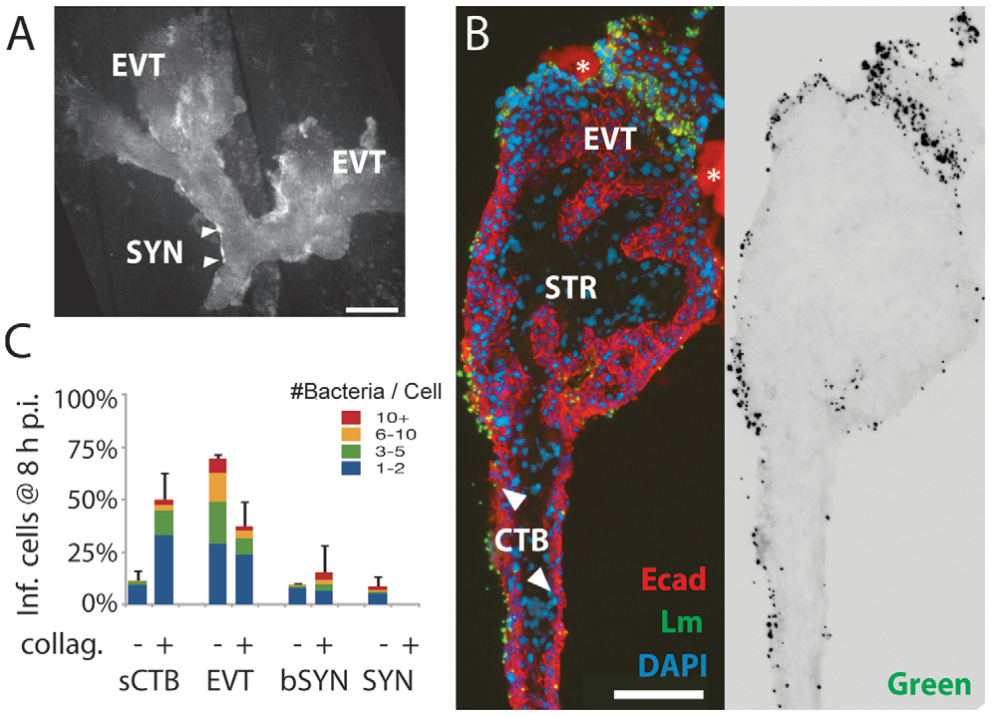
*L. monocytogenes* infects villous cytotrophoblasts when syncytiotrophoblast is removed. (**A**) Placental explant treated with collagenase-containing solution to degrade the syncytiotrophoblast (SYN). Treatment varies; some areas of syncytiotrophoblast remain (e.g. between arrowheads). All villi anchor to form extravillous cytotrophoblasts (EVT). Bar = 1 mm. (**B**) Left: histological section of enzymatically-treated villus arm, 8 h postinoculation (p.i.). No syncytiotrophoblast remains, permitting infection of both villous cytotrophoblasts (CTB) and extravillous cytotrophoblasts (EVT). Red = E-cadherin (Ecad). Green = *L. monocytogenes*. Blue = DAPI. Asterisk = Matrigel. Right: Green channel only, color inverted to show *L. monocytogenes* (solid black) with background fluorescence (faint grey) to show explant outline. Bar = 100 µm. (**C**) Distribution of infected cell types in enzymatically-treated explants compared to that in untreated explants at 8 h p.i. Here, sCTB refers to villous trophoblasts, which are subsyncytial in untreated explants but exposed after syncytiotrophoblast removal in enzymatically-treated explants. Each condition represents two sections from each of three explants. For syncytiotrophoblast (SYN) and basally accessible syncytiotrophoblast (bSYN), a “cell” was considered to be the area around a single nucleus, roughly the size of a cytotrophoblast. Bars = SEM.

If the syncytiotrophoblast is relatively resistant to infection, as the preceding data suggest, then removing it should provide *L. monocytogenes* new sites of invasion. We enzymatically degraded the syncytiotrophoblast by soaking the explants briefly in a collagenase-containing solution before plating [47]. Although the extent of the syncytiotrophoblast removal varied, many subsyncytial cytotrophoblasts were exposed and extravillous cytotrophoblasts increased (Figure 5A). As expected, permissive infections of enzymatically-treated explants allowed for new sites of infection (Figure 5B). The total number of infected cells increased (from an average of 78 in two sections to 228 with enzymatic degradation), and nearly half of the infected cells were now subsyncytial cytotrophoblasts, which express E-cadherin (Figure 5C; *p*<0.05 by Chi-square test).

### Cell-to-cell spread leads to infection of proximal extravillous cytotrophoblasts

Infection of the placenta by extracellular pathogens in the maternal bloodstream must be mediated by the interaction of pathogen virulence determinants, e.g. InlA with host cell receptors like E-cadherin. However, *L. monocytogenes* also traffics *in vivo* to the placenta in a gentamicin-resistant manner [40], presumably traveling inside phagocytic leukocytes [41]. We wanted to test whether cell-to-cell spread can mediate placental infection, and, if so, what sites are vulnerable.

**Figure 6:**
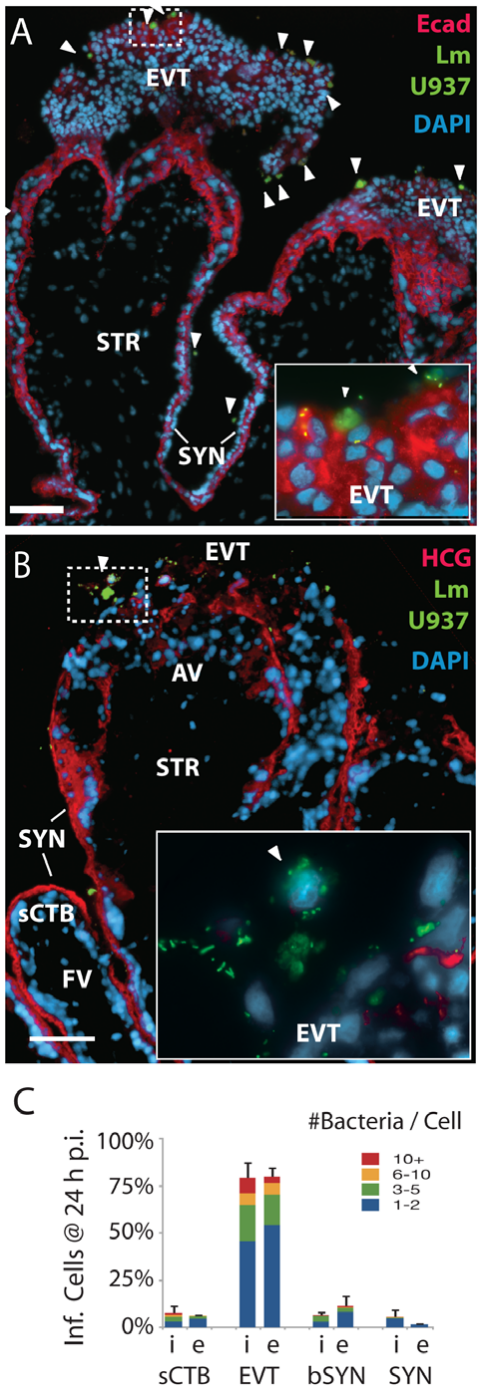
*L. monocytogenes* infects anchoring villi by cell-to-cell spread. (**A**) Histological sections of explant infected by *L. monocytogenes*-containing U937 macrophage-like cells loaded with green dye (arrowheads). *L. monocytogenes* are also stained green. Photos are 24 h post-inocculation (p.i.). Both bacteria and U937 cells localize primarily to the E-cadherin-expressing extravillous cytotrophoblasts (EVT). Red:  = Ecad. Blue = DNA. Bar = 100 µm. (**B**) Bacteria are excluded from syncytiotrophoblast (SYN) and subsyncytial cytotrophoblasts (sCTB). βHCG (red) is primarily expressed by syncytiotrophoblast. Only anchoring villi (AV) are infected, while floating villi (FV) covered in syncytiotrophoblast remain uninfected. Bar = 100 µm. (**C**) Localization of *L. monocytogenes* in explants when introduced in extracellular media (e) or by cell-to-cell spread from the intracellular compartment (i) of U937 cells (in the presence of gentamicin) at 24 h p.i. Each condition represents two sections in each of three explants. Bars = SEM.

We introduced a fluorescent live cell dye to macrophage-like U937 cells (differentiated to adherent cells with PMA) and then infected them with 10403S-sGFP *L. monocytogenes*. The infected cells were added to explants in the presence of gentamicin to prevent infection of the explant by extracellular bacteria. After 24 h transmission of *L. monocytogenes* from U937 cells to explants had occurred (Figure 6A-B). As with direct invasion, we found that *L. monocytogenes* infection by cell-to-cell spread from U937 cells to the placenta was largely confined to extravillous cytotrophoblasts at villous tips (Figure 6C). In fact, the cell populations infected were statistically indistinguishable from InlA-mediated infections after 24 h (*p* = 0.99 by chi-squared test). Invasive CTB express chemokines that attract cells of the monocyte lineage [48]–[50], and indeed we observed clusters of U937 cells around the extravillous cytotrophoblasts as early as 4–8 h post-inoculation (data not shown). Placental infection was not observed upon co-cultivation with U937 cells carrying ΔActA mutants, which are defective in cell-to-cell spread (data not shown).

### Bacterial dissemination occurs along subsyncytial cytotrophoblasts in anchoring villi

**Figure 7:**
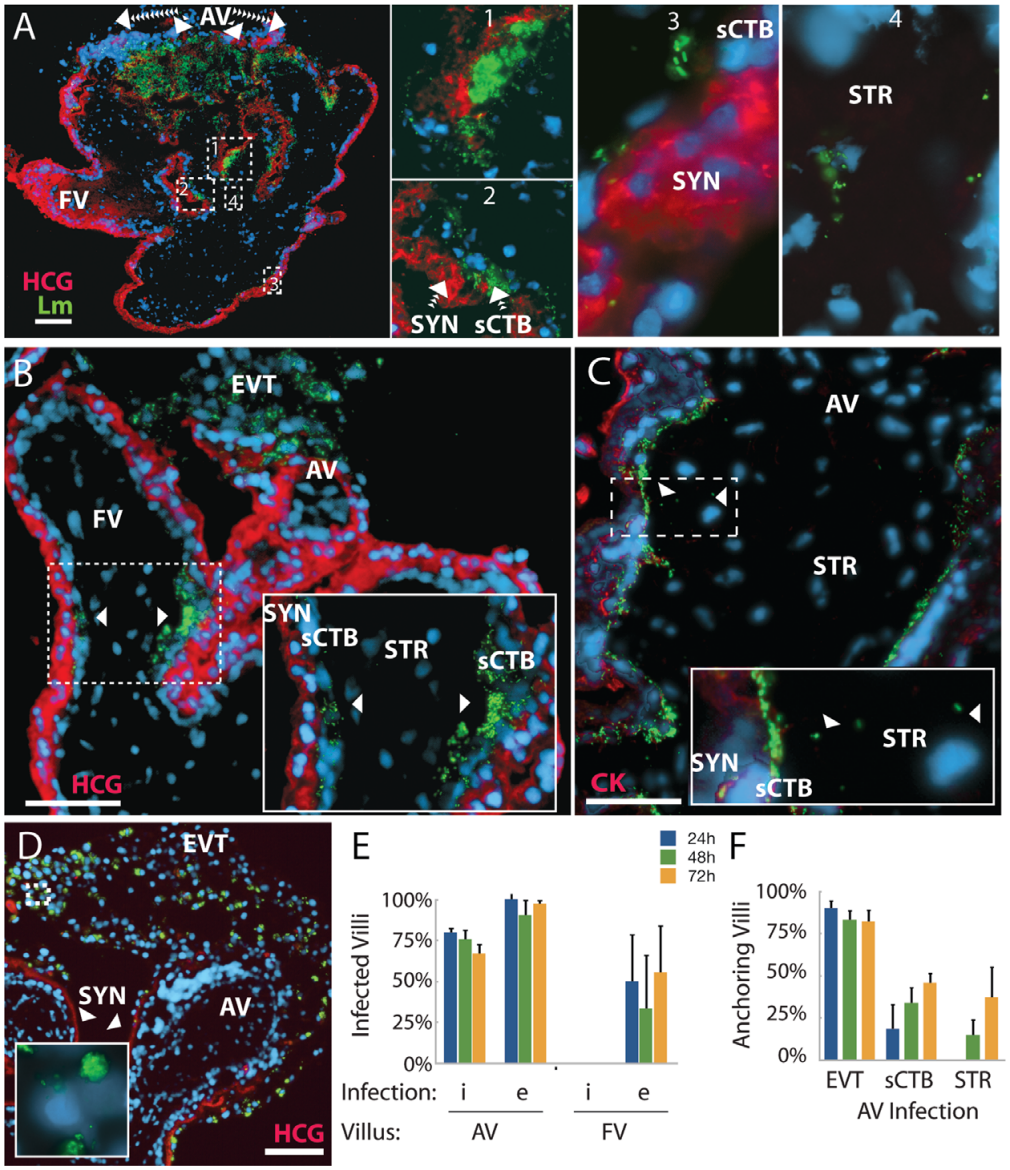
Infection progresses from extravillous cytotrophoblasts to stroma over 72 hours. (**A**) Histological section of explant permissively infected by *L. monocytogenes* at 72 h post-inoculation (p.i.)**.** Inset numbers correspond with right panels. Anchoring villi (AV) are major loci of infection, while floating villi (FV), which lack extravillous cytotrophoblasts (EVT), remain relatively uncolonized. Syncytiotrophoblast (SYN), indicated by βHCG (HCG, red) is still largely uninfected, with spread moving down the subsyncytial cytotrophoblasts (sCTB, insets 1 – 3) and occasionally crossing into stroma (STR, inset 4). (**B**) AV infection progresses from EVT toward fetus, while FV infection begins at the villus base. Here, a permissive infection shows dissemination from an anchoring villus (top center) to an FV, where bacteria circumnavigate the sCTB (arrowheads) while leaving SYN uninfected. (**C**) Infection from U-937 cells at 72 h p.i. shows bacteria concentrated in sCTB and bounded by the basement membrane that underlies them. A few bacteria have spread into STR (arrowheads, inset) where fetal capillaries are found. Red = cytokeratin (CK, stains cytotrophoblasts). (**D**) Explant permissively infected with ΔActA *L. monocytogenes*, which cannot spread from cell to cell. EVT are filled with bacteria (inset). Red = βHCG. (**A–D**) Green = *L. monocytogenes*, Blue = DAPI. Bar = 100 µm. (**E**) Percentage of AV and FV infected by *L. monocytogenes* introduced by cell-to-cell spread (i) or from extracellular media (e). FV infection was sporadic. (**F**) Dissemination of bacteria introduced by both means in AV. All infected AV contained bacteria in EVT. At later timepoints, infection of subsyncytial cytotrophoblasts and stroma rose. Stromal infection was not observed without sCTB infection. (**E–F**) Each condition represents two sections separated by at least 30 µm on the Z-axis in each of three placentas infected by each means. Bars = SEM.

Regardless of how *L. monocytogenes* was introduced, the dominant site of initial infection was the extravillous cytotrophoblast at the tip of anchoring villi. Multiple explants from a single placenta showed strikingly similar progression over the course of infection. In three out of six placentas studied, *L. monocytogenes* advanced significantly beyond the tips of anchoring villi (Figure 7). By 72 h post-inoculation, subsyncytial cytotrophoblast infection was common in anchoring villi while syncytiotrophoblast remained largely uninfected, suggesting that the syncytium not only resists cell-to-cell spread from macrophage-like cells but also from neighboring cytotrophoblasts (Figure 7A). While infected anchoring villi were always colonized at the distal tips, infection of floating villi always began at proximal junctures shared by anchoring villi. At times floating villi exhibited infection of cytotrophoblasts on both sides of the villus without syncytiotrophoblast infection, indicating that *L. monocytogenes* trafficked through the subsyncytial cytotrophoblasts (Figure 7B). Spread into the stroma was rare and presumably restricted by the basement membrane underlying subsyncytial cytotrophoblasts. Some stromal cells were infected at later timepoints (Figure 7C). In explants infected with ΔActA *L. monocytogenes*, bacteria remained in extravillous cytotrophoblasts (Figure 7D).

Overall, ∼75 – 100% of anchoring villi were infected (Figure 7E) and infection of subsyncytial cytotrophoblasts and stroma increased over 72 h (Figure 7F). In contrast, only 22% of explants exhibited any *L. monocytogenes* in floating villi (Figure 7E). Taken together, these results describe the cell-to-cell path *L. monocytogenes* follows in disseminating throughout placental explants over three days: from extravillous cytotrophoblasts of anchoring villi along lateral villous subsyncytial cytotrophoblasts and from there into floating villi and/or stroma, all while leaving the syncytiotrophoblast largely uninfected.

## Discussion

Pathogens present in the maternal bloodstream may colonize the placenta, causing infection, inflammation, and ultimately spontaneous abortion, preterm labor, and neonatal morbidity and mortality [51]. Many pathogenic microbes are found transiently in maternal blood. For example, the simple daily act of brushing teeth is associated with bacteremia [52],[53], and *L. monocytogenes* is ingested frequently by healthy adults [54]. Yet neither result in significant maternal-fetal infection the majority of the time. This is surprising considering that twenty percent of maternal blood can be found circulating freely in the placenta's intervillous space, where it bathes fetal villi that are covered by a syncytiotrophoblast whose surface area ranges from 3000 cm^2^ in the late first trimester to 125,000 cm^2^ at term [28]. Thus, it seems reasonable to hypothesize that the syncytium forms an extremely effective physical barrier against infection. In this study, we have conclusively shown that the syncytiotrophoblast is resistant to infection by *L. monocytogenes* and that extravillous cytotrophoblasts are the portal of entry.

For internalin-mediated infections the resistance of the syncytiotrophoblast can be reasonably explained by the tissue's lack of E-cadherin on the apical surface. When syncytiotrophoblast was infected, it was more likely to be basolaterally accessible syncytiotrophoblast, in which an E-cadherin expressing basolateral surface was exposed. Even for infections in uninterrupted syncytiotrophoblast, it remains possible that basolateral access was not apparent in the examined section but was available in an adjacent section. It is interesting to note that InlA and E-cadherin interactions mediating intestinal invasion are confined by anatomical and cellular barriers as well [55].

Even more surprising was the near absence of syncytiotrophoblast infection by cell-to-cell spread, either from U937 cells—which were observed near the syncytiotrophoblast—or from neighboring cytotrophoblasts at later timepoints. Three possible explanations exist: 1) the syncytiotrophoblast under-expresses unknown host molecule(s) required for cell-to-cell spread; 2) attachment of leukocytes to syncytiotrophoblast is insufficiently close in time or space for cell-to-cell spread to occur; or 3) the syncytiotrophoblast membrane is physically inhospitable to *L. monocytogenes*' actin-mediated protrusions. The last two are especially plausible when considering the profuse covering of branched microvilli on the apical surface [56]–[61]. The basal surface may also be girded against protrusion by the especially dense cytoskeletal network that is presumably required to resist cytosolic surface tension in the laterally vast syncytium [56]–[59]. Interestingly, interaction of the bacterial virulence factor InlC with human actin regulatory proteins has recently been shown to promote cell-to-cell spread by decreasing cortical tension, thereby enhancing the ability of motile bacteria to deform the plasma membrane into protrusions [62].

The syncytiotrophoblast may act as a general barrier. We have observed that *T. gondii* is not able to efficiently colonize the syncytium either (unpublished observations), and other groups have reported similar results for herpes simplex virus [63] and cytomegalovirus [64],[65].

**Figure 8:**
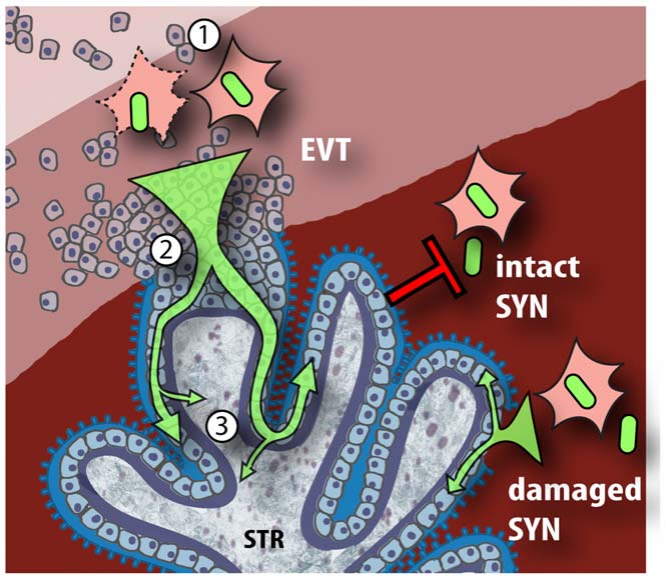
Model of *L. monocytogenes* mechanisms for breaching the maternal-fetal barrier. *L. monocytogenes* is subjected to multiple bottlenecks when infecting the placenta. Our data support extravillous cytotrophoblasts (EVT) as the primary portal of entry. First (1), relatively few *L. monocytogenes* reach the maternal decidua, carried by phagocytes. These can infect extravillous cytotrophoblasts by cell-to-cell spread, or by lysing the leukocyte and subsequently infecting extravillous cytotrophoblasts via InlA-E-cadherin interactions. (2) Extravillous cytotrophoblasts further winnow bacterial numbers by delaying the *L. monocytogenes* intracellular life cycle. If infection progresses, bacteria spread through subsyncytial cytotrophoblasts. (3) The basement membrane underlying these cells presents a third barrier, which few bacteria cross to invade the fetal stroma (STR). On the other hand, *L. monocytogenes* in the blood contacts only the syncytiotrophoblast (SYN), which is highly resistant to both internalin-mediated infection and intercellular spread. However, it is possible that sites of syncytial damage provide access to subsyncytial cytotrophoblasts. In vivo, such sites are rapidly covered by fibrinoid clots [28] that may present yet another physical barrier to infection.

Instead, we present evidence that extravillous cytotrophoblasts, normally not easily accessible from the intervillous space, are the dominant sites of *L. monocytogenes* colonization from both extracellular and intracellular compartments. Our results are in accord with Lecuit et al. showing that invasion of placental explants by extracellular *L. monocytogenes* depends on InlA, but our findings differ on the initial site of invasion (EVT versus SYN, see Figure 8). An important difference is our experimental set-up: we used first trimester placental explants instead of term placentas. Term placental organ cultures do not form anchoring villi after removal from the mother and are maintained in floating culture. Damage of the term explant syncytiotrophoblast has been reported as early as 4 h under selected culture conditions [66] and term explants cannot be used to evaluate extravillous cytotrophoblasts [67]. Thus, first trimester explants better represent the architecture of the maternal-fetal barrier *in vivo*.

However, placental organ cultures from all gestational ages omit the decidua and the maternal vessels. The later are remodeled by extravillous cytotrophoblasts, which differentiate into endovascular trophoblasts that replace the endothelium of the maternal spiral arteries and therefore are in direct contact with maternal blood. Endovascular trophoblasts do not express E-cadherin [32] but it may be possible that they are targets of cell-to-cell spread *in vivo*.

It has recently been postulated that the conjugated action of InlA and InlB leads to breaching of the maternal-fetal barrier [37]. This is particularly intriguing since InlA and InlB are in the same operon and expression of these invasion proteins is most likely co-regulated [16]. Disson et al. show a 10-fold reduction in invasion of the human intestinal cell line Caco-2 with *L. monocytogenes* strain EGDe, deficient in InlA or InlB, and an almost 100-fold reduction with the InlAB double deletion mutant [37]. Other groups using *L. monocytogenes* strains derived from 10403S have observed a 2–3-fold effect of InlB on intestinal invasion (Amieva, personal communication). We do not observe a difference between wt and ΔInlB in infection of early gestation placental organ cultures. It may be that the variability of the human placental explants is too high to resolve a potentially small effect of InlB on invasion of placental explants.

We did examine the role of cell-to-cell spread from infected U937 cells to the placenta, which seems highly relevant considering the importance of *Listeria*'s intracellular life cycle for virulence [68] and the published evidence that *L. monocytogenes* traffics between organs inside of cells [40],[41]. It is striking that extravillous cytotrophoblasts remain the primary portal of entry, leading us to hypothesize that access to extravillous cytotrophoblasts represents the first bottleneck for *L. monocytogenes* infection of the placenta (Figure 8). How can *L. monocytogenes* overcome this hurdle? Extravillous cytotrophoblasts are present in the decidua and are known to actively recruit macrophages, monocytes and natural killer cells [48]–[50]. Therefore, we postulate that *L. monocytogenes* reaches the placenta in maternal phagocytes that are recruited to the decidua where they infect extravillous cytotrophoblasts by cell-to-cell spread or internalin-mediated invasion.

Another striking finding was that extraordinarily high doses of *L. monocytogenes* were required to infect placental explants, and that *L. monocytogenes* growth rates were relatively slow, on average increasing only ∼10-fold over 24 h. However, we could distinguish two placental populations: in about half of the placentas *L. monocytogenes* did not grow, while in the other half bacterial numbers increased by ∼77-fold. Lecuit et al. used slightly higher doses and reported an increase of ∼100-fold over 24 h. But they also report having removed gentamicin from the culture medium at 2 h post-inoculation. Therefore, their 24 h CFU and histological assays may have included extracellular bacteria that escaped dying cells. In addition, we found little to no cell-to-cell spread at 24 h, while in most cell lines cell-to-cell spread begins as early as 4 h post-inoculation [21],[24]. In some placentas we observed that infection remained confined to the extravillous cytotrophoblasts for at least 72 h. It is intriguing that the sites of infection correlated with the host cell's proliferative capacity [69],[70], which may provide interesting avenues for future studies. The slow rate of intracellular bacterial growth and cell-to-cell spread suggest the possibility that extravillous cytotrophoblasts restrict the intracellular life cycle of *L. monocytogenes*, thus representing the second bottleneck in the placenta (Figure 8).

Once the placenta is infected, *L. monocytogenes* can spread to the fetus. One probable route of spread to the fetus is via the fetal capillaries in the villous stroma. Indeed, we observed low numbers of bacteria that had penetrated the basement membrane and infected the stroma suggesting that this is the third bottleneck *L. monocytogenes* encounters (Figure 8). It is interesting to note that although some bacteria were observed in syncytiotrophoblast at early timepoints, only anchoring villi acted as an origin of colonization.

Our model is consistent with previous findings *in vivo* that the guinea pig placenta is colonized by 10^4^ times fewer bacteria than maternal liver and spleen, and subsequently only 1 out of 10^4^ bacteria are able to spread from placenta to fetus [40]. It is also in agreement with the epidemiology of human listeriosis, which is a rare disease during pregnancy despite its ubiquity in the environment, as well as the observation that pregnant animals have to be inoculated with high doses of *L. monocytogenes* to observe consistent placental and fetal infection [37],[39],[40]. In addition, our results provide an explanation for the absent or minor phenotype the internalin mutants exhibit in multiple different pregnant animal models [37],[39],[40].

Although it may be attractive to describe the route taken by a pathogen as a single mechanism, we do not believe that this accurately reflects what occurs *in vivo*. There is mounting evidence that pathogens have evolved to exploit multiple strategies to breach the intestinal and blood-brain barriers [71],[72], and it is reasonable to expect the same of the maternal-fetal barrier. The mechanisms by which the placenta excludes most pathogens to generate the maternal-fetal barrier are poorly understood, but our results suggest that the syncytiotrophoblast plays a significant role. Given its extensive contact with the maternal blood, this important tissue may have evolved to exclude pathogens, and our model system offers a powerful way to probe the mechanisms by which this occurs. Pathogens that can breach the syncytiotrophoblast or exploit sites of syncytial damage may colonize the placenta via subsyncytial cytotrophoblasts. Our study of *L. monocytogenes*, a model pathogen that colonizes the placenta, strongly suggests that the placenta's most vulnerable site is the extravillous cytotrophoblast, where cells anchor the placenta in the maternal decidua but have little to no contact with maternal blood. This finding suggests a reason for the observation that almost all pathogens capable of crossing the maternal-fetal barrier are either facultative or obligate intracellular: dissemination in the blood is not enough.

## Methods

### Ethics statement

This study was conducted according to the principles expressed in the Declaration of Helsinki. The study was approved by the Institutional Review Board at the University of California, San Francisco, where all experiments were performed (H497-00836-28). All patients provided written informed consent for the collection of samples and subsequent analysis.

### Human tissue collection and culture

Placentas from elective terminations of pregnancy (gestational age 4 to 8 weeks) were collected and prepared as previously described [67]. Briefly, fragments from the surface of the placenta were dissected into 1–3 mm tree-like villi, placed on Matrigel (BD Biosciences, San Jose, CA) coated Transwell filters (Millipore, Bedirica, MA, 30-mm diameter, 0.4 µm pore size) and cultured in Dulbecco's modified Eagle's medium-F12 medium (DMEM-F12; 1∶1, vol/vol) supplemented with 20% fetal bovine serum (FBS, Fisher Scientific), 1% L-glutamine and 1% penicillin/streptomycin (Invitrogen, Carlsbad, CA). For surface area and perimeter measurements, cultured explants were photographed pre-infection on a Leica MZ16F stereomicroscope (Leica Microsystems, Wetzlar, Germany) using an Axiocam MR monochrome camera (Carl Zeiss, Munich, Germany). Measurements were made using ImageJ software (NIH, Bethesda, MD).

### Removal of syncytiotrophoblast

Syncytiotrophoblast was removed from villous trees as previously described [47]. Briefly, placental explants were soaked for 5–15 min in a solution containing Type IA collagenase (100,000 U), hyaluronidase (150,000 U), DNAse (120,000 U) and 0.1% BSA in PBS without divalent cations (UCSF Cell Culture Facility, San Francisco, CA). Explants were observed continuously via a dissecting microscope and when syncytiotrophoblast degradation was apparent they were transferred to Matrigel.

### Pathogen strains and growth conditions

The wild type strain of *L. monocytogenes* used in this study is 10403S [73]. Mutant strains included ΔInlA (DPL4405), ΔInlB (DPL4406), ΔInlAB (DPL4455) [30], ΔActA (DPL3078) [74], and sGFP-expressing 10403S *L. monocytogenes* (DH-L1039) [75]. EGDe *L. monocytogenes* (M. Loessner) was used for some experiments. Bacteria were cultured using brain heart infusion (BHI) broth or agar (Becton Dickenson Company, Sparks, MD).

### *L. monocytogenes* infections of placental explants

Intracellular growth assays of *L. monocytogenes* were performed as previously described [76] with following modifications: placental explants were incubated in antibiotic free media for 1 h prior to infection, 1×10^6^ bacteria/ml were added for 30 min and gentamicin (50 µg/ml) was added at 60 min post-inoculation. Gentamicin was subsequently maintained in the media, which was refreshed every 24 h. At specified times after infection, explants were removed from Matrigel and homogenized in 1 ml dH_2_O using a T25 digital Ultra-Turrax (IKA, Staufen, Germany). Aliquots were plated on BHI agar and grown at 37°C. For permissive infections explants were incubated with 2×10^7^ bacteria/ml for 5 h before adding gentamicin.

### Infection of explants by cell-to-cell spread

Human macrophage-like U937 cells (ATCC 1593.2 [77]) were grown in RPMI-1640 (UCSF Cell Culture Facility) containing 4500 mg/L glucose, 10% FBS and 1% penicillin/streptomycin (Invitrogen). Forty-eight hours prior to infection, cells were differentiated by addition of phorbol 12-myristate 13-acetate (PMA; concentration 18 nM; Sigma) to the medium. On the day of infection, cells were labeled with CellTracker Green CMFDA (Invitrogen) and infected with *L. monocytogenes* for 1 h at an MOI of 1∶1. Cells were washed once with PBS and lifted from culture plates by incubation in ice cold PBS without divalent cations for 5 min. U937 cells were resuspended in explant medium containing 50 µg/ml gentamicin, and 1×10^6^ cells per transwell were added to the explants. Every 24 h, fresh media containing gentamicin was added.

### Immunofluorescence and histology

Explants were removed at the times indicated and placed into vinyl cryomolds (Ted Pella, Redding, CA), then covered with optimal cutting temperature (OCT) media (Ted Pella) and flash-frozen. Histological slicing was performed using a Hacker-Slee cryostat. Glass slides with sections were incubated ∼5 min in acetone at 4°C. All antibody staining was conducted at room temperature. When dry, slides were soaked 60 min in blocking solution (1% bovine serum albumin (BSA, Sigma) in PBS), then rinsed and exposed to primary antibodies in 0.5% BSA/PBS. Slides were rinsed three times for 5 min each in 0.5% BSA/PBS, then secondary antibodies were added at the indicated concentrations and incubated for 60 min. After three rinses, coverslips were affixed over Vectashield mounting medium with DAPI (Vector Laboratories, Burlingame, CA). Uninfected explants did not stain with anti-*Listeria* antibodies. Primary antibodies: polyclonal rabbit *Listeria* O antiserum (1∶1000 Becton, Dickenson), monoclonal mouse anti-human cytokeratin 7 (1X, Clone OV-TL, Dako, Carpinteria, CA), monoclonal mouse anti-human E-cadherin (1∶200, Clone NCH-38, Dako), monoclonal mouse anti-human βHCG (1∶500, clone SPM105, Neomarkers, Fremont, CA) and monoclonal mouse anti-human EGFR (1∶250, Clone cocktail R19/48, Biosource, Camarillo, CA). Secondary antibodies: Alexa Fluor 594 goat anti-mouse IgG (1∶500) and Alexa Fluor 488 goat anti-rabbit IgG (1∶1000, both Invitrogen). All immunofluorescence conditions were compared to no-primary controls to ensure that non-specific binding did not occur.

Slides were viewed using an inverted TE2000-E microscope (Nikon, Tokyo, Japan) equipped with a 12-bit cooled CCD camera (Q Imaging, Surrey, Canada). Images were collected using Simple PCI software (Hamamatsu, Sewickley, PA). Counts of *L. monocytogenes* localization were made by tallying every infected cell in each section at 100X magnification.

### Confocal microscopy

Whole mount explants were prepared by rinsing explants with PBS and then soaking in 3% paraformaldehyde in PBS (Ted Pella) for 12 h at 4°C. Explants were then rinsed three times with PBS and suspended in 1∶100 Alexa Fluor 594 phalloidin and 1∶100 DAPI (both Invitrogen) for 24 h at 4°C. Explants were mounted onto glass slides in Vectashield and sealed under coverslips. Imaging was performed at the Nikon Imaging Center at UCSF using an upright Nikon C1 spectral confocal microscope equipped with 405, 488 and 561 nm lasers.

### Image processing for figures

Images were prepared using Photoshop and Illustrator (Adobe, San Jose, CA). RGB color hues were linearly adjusted for better CMYK printing but no non-linear alterations were performed.
